# Availability and prices of essential medicines for chronic diseases in older people in the Asia Pacific Region

**DOI:** 10.1186/2052-3211-8-S1-P3

**Published:** 2015-10-05

**Authors:** Tuan A Nguyen, Sun Qiang, Haipeng Wang, Krishna Undela, Agnes Vitry

**Affiliations:** 1 Quality Use of Medicines and Pharmacy Research Centre, School of Pharmacy and Medical Sciences, Sansom Institute, University of South Australia GPO, Adelaide, 5001, Australia; 2 Center for Health Management and Policy, School of Public Health, Shandong University, Shandong, 250100, China; 3 Department of Pharmacy Practice, JSS College of Pharmacy, JSS University, Karnataka, 570015, India

## Background

Little is known about the prices and availability of medicines for chronic diseases used by older people in the Asia Pacific Region. The objective of this study was to assess the availability and prices of essential medicines for chronic diseases in 11 countries, namely China, Fiji, India, Indonesia, Lao, Malaysia, Mongolia, the Philippines, Sri Lanka, Thailand and Vietnam. The study was carried out at an international level.

## Methods

A secondary analysis of medicine prices and availability data from the Health Action International's database on medicine prices, availability and affordability was undertaken in March - May 2015. Data on the availability and price of 15 medicines used for chronic diseases prevalent in the older population were obtained from facility-based surveys conducted in 11 countries between 2001 and 2013. Prices were converted into the base year of 2014. Patient prices were adjusted for inflation and purchasing power parity and procurement prices for inflation and official exchange rates. Data were analysed for lowest priced generic (LPG) and innovator brand (IB) products in both the public and private sectors.

## Results

The availability of medicines for chronic diseases was suboptimal across countries in the Region. The median availability of any medicine (IB or LPG) in the public sector was 35.5% compared with 56.7% in the private sector. Thailand and Indonesia had the highest levels of availability in the public sector (80% and 60.1% respectively) while in the private sector it was India and Fiji (90% and 83.4% respectively).

Countries in the Region paid 1.4 times the international reference price (IRP) to procure LPGs and 9.1 times the price for IBs. India and Fiji achieved low procurement prices (0.4 and 0.6 times IRP for LPGs) while the Philippines had the highest procurement prices for both IBs and LPGs. In general, patient prices were lower in the public sector than in the private sector (21.5 times IRP vs. 32.2 times for IBs and 6.6 times vs. 11.5 times for LPGs). In the public sector, Malaysia and India provided medicines free of charge while the Philippines charged the highest price.

## Conclusions

The availability and prices of medicines for chronic conditions were highly variable across the Asia Pacific Region. Medicines were more available in the private sector, but at an excessive price. Implementation of policies to improve the availability and reduce the prices of essential medicines for chronic diseases is needed.

